# Evaluation of adverse events in small‐breed dogs treated with maropitant and a single dose of doxorubicin

**DOI:** 10.1111/jvim.16439

**Published:** 2022-05-07

**Authors:** Fukiko Matsuyama, Kei Harada, Eri Fukazawa, Masanao Ichimata, Yuko Nakano, Tetsuya Kobayashi

**Affiliations:** ^1^ Japan Small Animal Cancer Center, Japan Small Animal Medical Center Tokorozawa‐shi Saitama Japan; ^2^ Veterinary Cancer Center, Hayashiya Animal Hospital Kyoto Japan

**Keywords:** adverse events, antiemetic drug, dose intensity, inappetence, small dogs

## Abstract

**Background:**

The recommended doxorubicin (DOX) dose for small dogs is 1 mg/kg. Recent data suggest that DOX‐induced gastrointestinal (GI) toxicosis can be reduced with maropitant treatment.

**Objectives:**

To investigate the incidence of adverse events (AEs) in small‐breed dogs administered a single 25 mg/m^2^ DOX followed by administration of maropitant (DOX25). The primary aim was to assess myelo‐ and GI toxicoses for 2 weeks after DOX administration. The secondary aim was to compare the incidence and grades of AEs found in the DOX25 group with a historical control group (DOX 1 mg/kg without administration of antiemetic or antidiarrheal medications).

**Animals:**

Nineteen small‐breed tumor‐bearing dogs.

**Methods:**

A prospective, observational study of tumor‐bearing dogs, weighing 5 to 10 kg, administered a single 25 mg/m^2^ dose of DOX IV, followed by administration of maropitant for the next 5 days.

**Results:**

Inappetence, vomiting, and diarrhea were found in 7/19, 2/19, and 6/19 of the DOX25 dogs, respectively. Neutropenia and thrombocytopenia was 12/19 and 3/19, respectively. Most AEs were grades 1 and 2, except for grades 3 and 4 inappetence and neutropenia in 3 and 4 dogs, respectively. Furthermore, febrile neutropenia occurred in 3/19 dogs in the DOX25 group. All AEs between the DOX25 and historical control groups were not significantly different.

**Conclusions and Clinical Importance:**

Vomiting and diarrhea were deemed acceptable with 25 mg/m^2^ DOX followed by maropitant treatment in 5 to 10 kg dogs; however, additional supportive care might be needed for dogs with inappetence and neutropenia.

**Abbreviations**
AEs: adverse events
DOX: doxorubicin
FN: febrile neutropenia
GI: gastrointestinal
NK1: neurokinin 1

## 1 INTRODUCTION

Doxorubicin (DOX) is a chemotherapeutic agent used to treat a variety of malignancies in dogs and cats.^1^, ^2^, ^3^, ^4^, ^5^, ^6^, ^7^, ^8^ Adverse events (AEs) associated with DOX include short‐term toxicoses, such as gastrointestinal (GI) and myelotoxicosis.^1^, ^8^ The incidence of DOX‐induced GI toxicosis is 24% to 64%.^1^, ^8^, ^9^ The most common signs related to GI toxicosis include inappetence, vomiting, diarrhea, and colitis that usually occur 3 to 5 days after DOX administration.^8^ Neutropenia is the primary consequence of myelotoxicosis with an incidence of 11% to 13.2%, usually occurring 5 to 10 days after DOX administration.^1^, ^8^, ^10^

Maropitant is an antiemetic used in dogs. It is an NK1 (neurokinin 1) receptor antagonist effective against chemotherapy‐induced vomiting.^9^, ^11^, ^12^ Administration of maropitant before cisplatin treatment is effective in cancer bearing dogs.^11^ Maropitant reduced incidence of vomiting in 59 tumor‐bearing dogs from 35% to 8%.

Doxorubicin is generally administered at 30 mg/m^2^ in dogs weighing ≥10 to 15 kg and at 1 mg/kg in dogs weighing ≤10 to 15 kg.^4^, ^13^ This body weight‐based dosing regimen is based on the observation that GI toxicosis and neutropenia (<1000 cells/μL) was more often seen in dogs weighing ≤10 kg than in those weighing ≥10 kg after the administration of 30 mg/m^2^ DOX.^14^ Depending on body weight, the dose calculated with mg/kg could actually be lower than that calculated with mg/m^2^ and therefore might not be a sufficient therapeutic dose. Without an appropriate phase 1 clinical trial evaluating DOX dosing in small dogs, few attempts have been made to increase dosing in small dogs over concerns about AEs, especially GI toxicosis.

This study aimed to prospectively investigate the incidence of GI and bone marrow AEs in dogs weighing 5 to 10 kg, treated with the initial single DOX dose at 25 mg/m^2^, followed by maropitant treatment (DOX25). The secondary aim was to compare the incidence and grades of AEs found in the DOX25 group with a historical control group (DOX 1 mg/kg without antiemetics and antidiarrheals). We hypothesized that the combined use of maropitant with DOX would allow the DOX dose to be increased in small dogs.

## 2 MATERIALS AND METHODS

### 2.1 Study animals

This prospective observational study included dogs weighing 5.0 to 10.0 kg that were presented to the Japan Small Animal Cancer Center at the Japan Small Animal Medical Center between May 2012 and September 2017. Dogs were included in the study if they were to receive an initial DOX dose of either a single agent or as part of a multiagent protocol for the treatment of various malignancies diagnosed by cytology, histopathology, or diagnostic imaging (ie, cardiac hemangiosarcoma). Dogs were excluded from the study if they had an earlier history of DOX treatment, received any antiemetic or antidiarrheal agents after DOX treatment, or had preexisting GI toxicosis before DOX administration, such as inappetence, vomiting, and diarrhea, or had a life expectancy of less than 1 month. However, we did not restrict drugs that were related to an underlying disease and had been prescribed before DOX administration or drugs prescribed for febrile neutropenia (FN) prophylaxis. Complete blood count, serum chemistry panels, thoracic radiographs, and abdominal ultrasound imaging were performed in all dogs at the initial visit for staging. Before the study, written informed consent was obtained from all owners.

### 2.2 Study design

All dogs were administered a 25 mg/m^2^ DOX dose (Doxorubicin Hydrochloride; ADRIACIN Injection 10, Aspen Japan, Tokyo, Japan) by IV infusion over 30 to 60 minutes without any premedication. Doxorubicin was diluted in 25 to 100 mL physiologic saline. Maropitant (Maropitant citrate monohydrate, Cerenia, Zoetis Japan, Inc, Tokyo, Japan) was administered PO once a day at a dose of 2 mg/kg for 5 days from the day after DOX administration. To assess the general condition of the dogs, owners were asked to record their dog's activity level, appetite, and water intake throughout the day on a 10‐point scale. We asked the owners to measure body temperatures by rectal temperature, heart rates by palpable heartbeat in the left thorax or femoral artery, and respiratory rates by abdominal movement with breathing. All measurements were taken at rest and at home twice a day. The measured items were written on a sheet and submitted weekly. For GI toxicosis evaluations, we asked about the severity of vomiting (frequency and duration per day) and diarrhea (a 4‐level severity scale about the frequency and duration per day) for 2 weeks after DOX administration. To assess myelotoxicosis, CBC was performed 7 and 14 days after DOX administration to determine the incidence of neutropenia and thrombocytopenia. Febrile neutropenia was defined as a body temperature of ≥39.2°C and neutrophil count of ≤2500 cells/μL,^15^ and its incidence during the study period was recorded. Gastrointestinal toxicosis and myelotoxicosis were graded according to the Veterinary Cooperative Oncology Group Common Terminology Criteria for Adverse Events (VCOG‐CTCAE v1.1).^16^ Treatments for AEs were performed at the discretion of attending veterinarians and was not standardized. In addition, the incidences of GI toxicosis and myelotoxicosis were compared with those in the historical control group.

### 2.3 Historical control group inclusion criteria

The historical control group consisted of dogs weighing 5 to 10 kg that were presented to the Japan Small Animal Cancer Center at the Japan Small Animal Medical Center, or Kobayashi Animal Hospital, Saitama, between January 2003 and December 2009. These dogs received DOX at 1 mg/kg to treat various malignancies and received no antiemetics or antidiarrheals after DOX administration. However, we did not restrict drugs related to an underlying disease and had been prescribed before DOX administration, or drugs prescribed for FN prophylaxis. We obtained signalment, tumor types, concomitant medications, presence of vomiting or diarrhea, and CBC on days 7 and 14 after the DOX administration.

### 2.4 Statistical analysis

For statistical comparison between the DOX25 and historical control groups, the Wilcoxon rank‐sum test was used for age and body weight. The Chi‐square test or Fisher's exact test was used for sex ratios and AE incidences. Differences were considered significant at a *P* < .05. All statistical analyses were performed using Stata Statistical Software Ver. 14.2 (StataCorp, College Station, Texas).

## 3 RESULTS

### 3.1 Study demographics

A total of 19 dogs were included in the DOX25 group. The median age was 10 years (range, 5‐14 years). There were 3 intact and 6 castrated males and 2 intact and 8 spayed females. There were 9 Miniature Dachshunds, 2 Miniature Schnauzers, and 1 of a different breed. The mean body weight was 6.5 kg (range, 5.03‐10.0 kg). The most common tumor type was high‐grade lymphoma (multicentric = 4, hepato‐splenic = 2), followed by hemangiosarcoma (right atrium = 1, subcutaneous = 2, spleen = 1), transitional cell carcinoma of the bladder (n = 3), subcutaneous soft tissue sarcoma (n = 2), sarcoma (spleen = 1, liver = 1), mandibular osteosarcoma (n = 1), and mammary gland adenocarcinoma (n = 1). As for the disease status at the time of DOX administration, 4 dogs with multicentric lymphosarcoma (LSA) and 1 dog with hepato‐splenic LSA had complete remission, and 1 dog with hepato‐splenic lymphoma did not have abdominal ultrasonography or a cytologic examination immediately before the DOX administration because of a complete resolution of clinical signs. For tumors other than lymphoma, 4 dogs had gross lesions, and 9 had microscopic lesions at the time of DOX administration. Six dogs received DOX as part of a multidrug protocol, and 13 dogs received DOX as a single agent. The mean and median doses in the DOX25 group were 8.9 and 8.8 mg, respectively. If these dogs had been administered 1 mg/kg dose, the mean and median dose would have been 6.7 and 6.5 mg, respectively. Compared with DOX at 1 mg/kg, the mean dose intensity of DOX at 25 mg/m^2^ was 1.32 times higher and the median was 1.35 times higher. Complete blood count on day 7 were performed on day 7 after the DOX administration in 14 dogs and on day 7 ± 1 in the remaining 5 dogs for owner convenience. Complete blood count on day 14 were performed on day 14 in 11 dogs and on day 14 ± 2 in the remaining 8 dogs for the same reason.

### 3.2 Adverse events

The incidence of signs of GI toxicosis in the DOX25 group was 7/19 for inappetence, 2/19 for vomiting, and 6/19 for diarrhea. Most GI toxicosis assessments were grades 1 and 2, except for grade 3 inappetence in 3 dogs (Table 1). The incidence of neutropenia and thrombocytopenia in the DOX25 group was 12/19 and 3/19, respectively. The majority of neutropenic events were grades 1 and 2, except for grades 3 and 4 neutropenia observed in 1 and 3 dogs, respectively (Table 2). The incidence of FN in the DOX25 group was 3/19, and FN was observed in all 3 dogs with grade 4 neutropenia. All dogs with FN recovered without any problems.

**TABLE 1:** A comparison of incidence of signs of gastrointestinal toxicosis between the study and historical control group dogs

	DOX 25 mg/m^2^, n = 19	%	DOX 1 mg/kg, n = 18 (17^a^)	%	Odds ratio	95% CI	*P*‐value
Inappetence	7	36.8	7	41.2	0.8	0.2‐3.9	1
G1	2	10.5	3	17.6			
G2	2	10.5	1	5.9			
G3	3	15.8	3	17.6			
G4	0	0	0	0			
Vomiting	2	10.5	5	27.8	0.3	0.03‐2.3	.23
G1	2	10.5	4	22.2			
G2	0	0	1	5.6			
G3	0	0	0	0			
G4	0	0	0	0			
Diarrhea	6	31.6	9	50	0.46	0.1‐2.1	.32
G1	5	26.3	7	38.9			
G2	1	5.3	2	11.1			
G3	0	0	0	0			
G4	0	0	0	0			

^a^ One dog in the historical control group had a gastrostomy tube and was excluded from the analysis of inappetence.

**TABLE 2:** A comparison of incidence of signs of cytopenia between the study and historical control group dogs

	DOX 25 mg/m^2^, n = 19	%	DOX 1 mg/kg, n = 18	%	Odds ratio	95% CI	*P*‐value
Neutropenia	12	63.2	6	33.3	3.4	0.716.5	.1
G1	7	36.8	4	22.2			
G2	1	5.3	2	11.1			
G3	1	5.3	0	0			
G4	3	15.8	0	0			
Thrombocytopenia	3	15.8	1	5.6	3.2	0.2177.0	.6
G1	1	5.3	0	0			
G2	2	10.5	1	5.6			
G3	0	0	0	0			
G4	0	0	0	0			
Febrile neutropenia	3	15.8	1	5.6	3.2	0.2177.0	.6

### 3.3 Concomitant treatments

Aside from maropitant treatment, the following concomitant medications were used after DOX administration: drugs given as disease‐related were prednisolone (n = 5), firocoxib (n = 4), meloxicam (n = 1), enalapril (n = 1), and levothyroxine (n = 1). Famotidine was prescribed to 6 dogs receiving long‐term treatments with prednisolone or NSAIDs. The drugs given for FN prophylaxis were enrofloxacin (n = 16), cephalexin (n = 1), fosfomycin (n = 1), and ofloxacin (n = 1). One of the 2 dogs that had vomiting was receiving enrofloxacin as a concomitant medication, but the other dog had not received any concomitant medication at the time vomiting was observed. Five of the 6 dogs that had diarrhea received either enrofloxacin, ofloxacin, or fosfomycin, and 3 of the 6 dogs received either famotidine, firocoxib, or prednisolone concomitantly (partially overlapping). One dog did not receive any concomitant medication at the time diarrhea was observed. Six of the 7 dogs that had inappetence were receiving either enrofloxacin or ofloxacin, and 3 of the 7 were receiving famotidine, firocoxib, or meloxicam (partially overlapping). One dog did not receive any concomitant medication at the time inappetence was observed. On the other hand, 10 dogs that did not experience vomiting, diarrhea, or inappetence received either enrofloxacin or cephalexin in all cases, and famotidine, prednisolone, firocoxib, enalapril, or levothyroxine in 7 dogs (partially overlapping). Enrofloxacin was administered to 2 of the 3 dogs with FN and ofloxacin in 1 dog. Enrofloxacin was administered to 2 of the 3 dogs with FN and ofloxacin in 1 dog (Table S1).

### 3.4 Demographics of the historical control group

A total of 18 dogs were included in the historical control group. The median age was 9.5 years (range, 3‐14 years). There were 7 intact and 4 castrated males and 4 intact and 3 spayed females. There were 6 Shih Tzu, 4 Miniature Schnauzers, 2 Miniature Dachshunds, 2 mixed dogs, and 1 of a different breed. The mean body weight was 6.4 kg (range, 5.2‐9.5 kg). There were no significant differences in age, sex, or body weight between the DOX25 and historical control groups (Table 3). The tumor types included high‐grade lymphoma (multicentric = 5, GI = 1, cutaneous = 2, hepatic = 1, oral lymphoma with bone metastasis = 1), carcinoma (oral undifferentiated adenocarcinoma or squamous cell carcinoma = 1, cutaneous apocrine adenocarcinoma = 1), mammary gland adenocarcinoma (n = 2), nasal adenocarcinoma (n = 1), hepatic liposarcoma (n = 1), fibrous histiocytic nodule (n = 1), and subcutaneous undifferentiated sarcoma (n = 1). As for the disease status at the time of DOX administration, 9 dogs with lymphoma had complete remission, and 1 dog with cutaneous lymphoma had gross disease. For tumors other than lymphoma, 6 dogs had gross lesions and 2 had microscopic lesions at the time of DOX administration. In the historical control group, 8 dogs received DOX as a single agent, and 10 dogs received DOX as a part of a multidrug protocol. Doxorubicin was administered as the initial dose in all cases. The following concomitant medications were used after DOX administration: drugs given as disease‐related were prednisolone (n = 6), piroxicam (n = 3), meloxicam (n = 1), thalidomide (n = 2), ursodeoxycholic acid (n = 1), and enalapril (n = 3). Famotidine was prescribed in 6 dogs receiving long‐term treatment with prednisolone or NSAIDs. The drugs given for FN prophylaxis were cephalexin (n = 3), enrofloxacin (n = 1), minocycline (n = 1), and ampicillin (n = 1). In the historical control group, all dogs had CBC on day 7 ± 1 after DOX administration. Thirteen dogs had CBC on day 14 ± 1, 2 on day 11, and 1 on day 12 after DOX administration. Two dogs did not have CBC on day 14. One dog had a gastrostomy tube and was excluded from the inappetence analysis.

**TABLE 3:** A comparison of signalments between the study and historical control group dogs

	DOX 25 mg/m^2^, n = 19	DOX 1 mg/kg, n = 18	*P*‐value
Age, years IQR (range)	10.0 (8‐13)	9.5 (6.5‐12.2)	.40^a^
Weight, kg IQR (range)	6.5 (5.4‐8.0)	6.4 (6.0‐7.7)	.58^a^
Gender			
Male	9	11	.51^b^
Female	10	7	

^a^ Wilcoxon rank‐sum test.

^b^ Chi‐square test.

### 3.5 Comparison of AEs with the historical control group

The incidence of GI toxicosis in the historical control group was as follows: 7/17 for inappetence, 5/18 for vomiting, and 9/18 for diarrhea. There was no significant difference compared with the DOX25 group for any GI toxicosis (Table 1). The incidence of neutropenia and thrombocytopenia was 6/18 and 1/18, respectively, and were grades 1 and 2 in all cases. There was no significant difference compared with the DOX25 group for myelotoxicosis (Table 2). The incidence of FN in the historical control group was 1/18, and the dog had a grade 2 neutropenia. There was no significant difference in the incidence of FN between the 2 groups (*P* = .60; Table 2).

## 4 DISCUSSION

Our results show that all vomiting and diarrhea was grades 1 and 2 range and was clinically acceptable when DOX 25 mg/m^2^ was administered in combination with maropitant to dogs weighing 5 to 10 kg. On the other hand, inappetence was seen in 36.8% of the dogs, 3 of which were grade 3. In the historical control group, inappetence was also seen in 41.2% of the dogs, 3 of which were grade 3. These results suggest that inappetence after DOX administration should be a concern at any dose. Regarding neutropenia in the DOX25 group, 3 dogs had grade 4 neutropenia, which developed into FN in all of these dogs. This suggests that close monitoring, including temperature pulse respiration measurements at home and supportive care, such as prescription of prophylactic antibiotics, are needed when DOX 25 is administered to dogs weighing 5 to 10 kg.

We showed that vomiting in small dogs weighing 5 to 10 kg, treated with DOX at 25 mg/m^2^ with concomitant maropitant, was acceptable. The incidence of vomiting is proportional to dose intensity,^8^ meaning that higher dose intensities are associated with greater incidence of vomiting. Therefore, because the incidence of vomiting, which was expected to be higher in the DOX25 group, was actually comparable with that in the DOX 1 mg/kg (historical control) group, it might suggest that maropitant administration with DOX could reduce vomiting at this higher DOX dose in dogs weighing 5 to 10 kg. In the present study, the incidence of vomiting was 10.5% (2/19), and all grades of vomiting were mild. In addition, we did not find a significant difference in the incidence of vomiting between the DOX25 and historical control groups DOX 1 mg/kg without antiemetics or antidiarrheals. However, the results should be interpreted with caution, as the incidence in the DOX25 group might not have been completely comparable with that of the historical control group as a type II error because of small sample sizes. Also, the lack of a significant difference does not mean that they are equivalent.

Our results suggest that maropitant could also reduce diarrhea caused by DOX. The concomitant use of maropitant with DOX could also reduce diarrhea and vomiting.^9^ Neurokinin 1 receptors, where maropitant binds, exist not only in the central chemoreceptor trigger zone but also in the intestinal tract.^17^, ^18^ Other studies have shown that substance P plays a significant role in the development of diarrhea in ulcerative colitis and Crohn's disease by binding to NK1 receptors.^19^, ^20^ These facts suggest that NK1 receptor antagonists, such as maropitant, can also act on peripheral receptors to inhibit the development of diarrhea. The incidence of diarrhea in the present study was comparable to that reported previously (33%)^9^ but showed no significant difference between the DOX25 and historical control groups. In any case, all diarrheal events observed in the 6 dogs of the present study were grades 1 and 2 and considered clinically manageable.

Our data showed that maropitant effectively reduced the incidence of DOX‐induced vomiting but did not improve DOX‐induced inappetence. The incidence of inappetence after treatment with DOX with concomitant maropitant was 36.8%. In addition, grade 3 inappetence was observed in both the DOX25 and historical control groups, suggesting that it might be necessary to treat inappetence at any dose after DOX administration. The effects of ondansetron, maropitant, and metoclopramide on cisplatin‐induced vomiting and nausea in dogs were reported in a single‐blind crossover study.^21^ Ondansetron, maropitant, and metoclopramide were administered to 8 dogs each immediately after cisplatin and compared the incidence of vomiting and nausea between treatment groups. The results showed that both ondansetron and maropitant effectively reduced vomiting, whereas only ondansetron was effective in improving nausea. Capromorelin is a ghrelin receptor agonist approved by the Food and Drug Administration as an appetite stimulant in dogs. The efficacy of capromorelin for dogs with anorexia was evaluated in a prospective, randomized, masked, placebo‐controlled clinical study and confirmed its appetite‐stimulating effects.^22^ In addition, mirtazapine has been used as a treatment for inappetence in dogs.^23^ It is thus important to consider the concomitant use of maropitant with other antiemetics, such as ondansetron, and appetite‐stimulating agents, such as capromorelin or mirtazapine for the management of DOX‐induced inappetence and nausea.

In the DOX25 group, 4 dogs had grade 3 or higher neutropenia, and all 3 dogs with grade 4 neutropenia developed FN, suggesting that more aggressive strategies for neutropenia may be needed when administering DOX at 25 mg/m^2^ to dogs weighing 5 to 10 kg. Different DOX doses in dogs and reported that treating dogs weighing ≤10 kg with DOX at a dose of 30 mg/m^2^ resulted in severe neutropenia.^14^ Based on this observation, it has been recommended that dogs weighing ≤10 kg be treated with DOX at a dose of 1 mg/kg. These results suggest that when administering DOX at 25 mg/m^2^ to dogs weighing 5 to 10 kg, appropriate supports should be taken for the possible occurrence of FN, such as prescribing prophylactic antibiotics and carefully monitoring animal conditions at home. In our study, although there was no significant difference in neutropenia compared with the historical control group, the results should be interpreted with caution as a type II error because of small sample sizes could have occurred.

Limitations to this study include the nonrandomized design and the fact that the study only evaluated AEs after the initial DOX administration but not after additional doses of DOX at 25 mg/m^2^. The tumor types and chemotherapy protocol (ie, DOX as a monotherapy or as a part of a multiagent protocol) were not standardized in our study, and the results might have been different if the protocols had been standardized. Moreover, we did not restrict drugs that were related to the underlying disease and had been prescribed before DOX administration or drugs prescribed for FN prophylaxis. These drugs might have been confounding factors for GI toxicosis. Because of the nature of the historical control group, the staging before DOX, monitoring after DOX, description of vomiting, diarrhea, and inappetence were not as complete as those in the DOX25 group. It is generally accepted in clinical studies to compare study groups with a group of historical control animals; however, the drug doses should be the same between the 2 groups. Before the start of the present study, a 1 mg/kg dose was used in dogs weighing less than 10 kg at our center and the Kobayashi Animal Hospital. Therefore, it was not possible to include dogs weighing >10 kg that were treated with a DOX 25 mg/m^2^ dose in the historical control group.

In conclusion, although a DOX dose of 25 mg/m^2^ combined with maropitant treatment was considered acceptable in terms of the incidence of vomiting and diarrhea, the fact that FN was observed in 15.8% of dogs suggests that additional management for neutropenia are needed when administering DOX at this dose. Although maropitant was effective in reducing vomiting, it did not show sufficient efficacy against inappetence. Therefore, it is important to consider additional management for inappetence, such as the concomitant use of ondansetron, mirtazapine, and capromorelin.

## CONFLICT OF INTEREST DECLARATION

Zoetis Japan, Inc. provided maropitant tablets (Cerenia) and partially paid the translation fee. The Japanese Foundation of Veterinary Specialist Scholarship (JFVSS), represented by T. Kobayashi, received a sponsorship from Zoetis Japan, Inc. in the past 5 years. Zoetis Japan Inc. did not contribute to the analysis of the results. No other authors have a conflict of interest.

## OFF‐LABEL ANTIMICROBIAL DECLARATION

Some dogs in this study received cefalexin, fosfomycin, metronidazole, ampicillin, ofloxacin, and minocycline as part of their treatment to prevent febrile neutropenia. In Japan, cefalexin, ampicillin, and ofloxacin have been approved for use in humans and animals, and both products were used in this study. The other drugs are not licensed in Japan for use in dogs. We chose to use these drugs in the dogs of this study because the drugs are considered safe, and we deemed them necessary for treatment.

## INSTITUTIONAL ANIMAL CARE AND USE COMMITTEE (IACUC) OR OTHER APPROVAL DECLARATION

Approved by the hospital board of the Japan Small Animal Medical Center (approved on 1 May 2012).

## HUMAN ETHICS APPROVAL DECLARATION

Authors declare human ethics approval was not needed for this study.

## Supporting information


**Supplemental Table S1** Adverse event descriptions
