# Supplementary material for: Evaluation of adverse events in small‐breed dogs treated with maropitant and a single dose of doxorubicin
Source: J Vet Intern Med. 2022 May 7;36(4):1409–15. doi: 10.1111/jvim.16439 (PMC9308424; doi:10.1111/jvim.16439)
Supplement: Supplementary file 1 — Supplemental Table S1 Adverse event descriptions [file JVIM-36-1409-s001.pdf]

Supplemental Table. Adverse event descriptions.

No.	Concomitant treatments	Inappetence	Vomiting	Diarrhea	FN
1	enrofloxacin	1 (grade3)	1	1	1
2	enrofloxacin	1 (grade2) *	1 *	1 *	
3	ofloxacin, famotidine, firocoxib	1 (grade3)		1	1
4	enrofloxacin	1		1	
5	enrofloxacin	1			1
6	meloxicam, enrofloxacin	1 (grade2)			
7	enrofloxacin	1 (grade3)			
8	enrofloxacin, prednisolone			1 (grade2)	
9	firocoxib, famotidine, fosfomycin			1	
10	enrofloxacin				
11	prednisolone, famotidine, enrofloxacin, levothyroxine				
12	enrofloxacin				
13	prednisolone, famotidine, enrofloxacin				
14	enrofloxacin				
15	firocoxib, cephalexin				
16	prednisolone, enrofloxacin, famotidine				
17	enrofloxacin, enalapril				
18	prednisolone, enrofloxacin				
19	firocoxib, famotidine, enrofloxacin				

* Before administration of enrofloxacin
